# Biodeterioration effects of three *Aspergillus* species on stucco supported on a wooden panel modeled from Sultan al-Ashraf Qaytbay Mausoleum, Egypt

**DOI:** 10.1038/s41598-023-42028-x

**Published:** 2023-09-14

**Authors:** Hala A. M. Afifi, Maisa M. A. Mansour, Alyaa G. A. I. Hassan, Mohamed Z. M. Salem

**Affiliations:** 1 Conservation Department, Faculty of Archaeology, Cairo University, Giza, 12613 Egypt; 2 Forestry and Wood Technology Department, Faculty of Agriculture (El-Shatby), Alexandria University, Alexandria, 21545 Egypt

**Keywords:** Microbiology, Environmental sciences, Materials science

## Abstract

This study focuses on the magnificent decoration of a painted and gilded wooden panel with signs of fungal biodeterioration caused by *Aspergillus* species in the Mausoleum of Sultan al-Ashraf Qaytbay, Cairo, Egypt. Numerous spectroscopic analyses and investigation techniques, including Scanning Electron Microscope Equipped with Energy Dispersive X-ray analysis (SEM–EDX), Fourier Transform Infrared analysis (FTIR), and X-Ray Diffraction (XRD) have been used to study the materials that comprise this painted and gilded wooden panel composition. *Aspergillus niger*, *A. flavus*, and *A. terreus* were recognized as isolated fungi, and their accession numbers are OQ820164, OQ820163, and OQ820160, respectively. The findings showed that the wooden support is of pinewood (*Pinus halepensis*), the white priming layer on top of the wooden support was identified as gypsum, the blue paint layer has been proposed to be Azurite, Au (gold) was the primary composition of the gilding layer, while Pb (lead) was detected in some spots, suggesting the use an alloy of gold with lead, and finally, animal glue was the bonding medium. Based on these findings, mimic samples with identical substrates and structural components have been designed, and the biodeterioration signs by the growing of the three *Aspergillus* species—*A. niger*, *A. flavus* and *A. terreus* were evaluated via SEM and color change. However, *A. niger* was discovered with density growth on surfaces of pinewood, gypsum, and Azurite and with less growth on the gilding layer after 6-month incubation. This contrasts with *A. terreus* and *A. flavus*, which had greater density growth on Azurite and stucco than on pinewood and less growth on the gilding layer. The used analytical methods with detailed analyses revealed the novelty and significant future aspects of the conservation of the painted and gilded wooden panel. Particularly given that this location is used for prayer and is crowded with people five times a day, which increases the accumulation of fungi and negatively affects both the historic Mosque and the worshippers' health.

## Introduction

The Burji or Circassian Mamluk period, which controlled Egypt from 1382 to 1517 AD, is characterized by its elaborate funeral structures, including the funerary complex built by Sultan al-Ashraf Qaytbay in the Northern Cemetery of Cairo (completed in 1474 AD). The mosque, madrasa, and mausoleum of Sultan al-Ashraf Qaytbay are frequently considered as some of the most exquisite structures and remarkable monuments of late Mamluk architecture (879 AH/1474 AD)^1–3^. As Sultan Qaitbay was a patron of architecture throughout his lengthy reign, this complex, along with other structures constructed by Sultan Qaitbay in Cairo, is among the most essential and technically significant structures in the city, especially the woodwork, which is some of the most distinctive and innovative designs of Mamluk architecture^4–6^. The design of the elements of this complex is notable for effectively meeting the psychological, physical, social, environmental and religious requirements of the period and location in which they appear. The Islamic nature of the structure undoubtedly has a significant impact on its components, as well as on its overall design and decoration^7–11^.

The wooden panel is found above a wooden door in one of the south-facing walls of the Qibla Iwan. The door opens to the mausoleum's dome, which is an obvious indication of Andalusian architectural influences. The splendor and originality of the complex's architectural components set it apart. Despite the usage of themes that have previously been featured in Mamluk buildings, the dome is original in its design^12^.

During the Islamic era, many buildings in Egypt were lavishly decorated with stucco ornamentation, which also took on significant architectural decorative features like wall friezes, niches, and stained-glass windows^8^. It was used on a variety of substrates, including linen, papyrus, wood, limestone, and sandstone^9–11^.

The chemical composition of the painted and gilded wooden panel was identified via spectroscopic investigations. Through spectral analyses, a mixture of gypsum and huntite was employed as the principal paint for Qajar paintings in Iran^13^. The decorative paints used in the structure were copper powder coated with gold (copper and zinc), red lead, Prussian blue and green. In order to produce color tones, white lead was also employed^13^. The potential novel function of strontium in identifying natural gypsum was examined using X-ray fluorescence measurements in various mineral samples of gypsum and alabaster as well in tempera, fresco and Egyptian paintings^14–16^. Azurite, malachite, vermilion, and gaseous black are among the paints used in the metal frescoes of the decorative materials on the ceiling of the chapter house of the Seville Town Hall. Lime mortar and ground dolomite are also employed throughout^17^.

A coffin from the Saqqara excavation's coating layer is made of two layers of gypsum with calcite, quartz, and dolomite inclusions and is painted with yellow ochre and carbon black ^18^. By Raman microspectroscopy combined with scanning electron microscopy, the wall paints used by medieval painters to paint the Madonna and Child with Saints Ambrogio Lorenzetti (early fourteenth century; St. Augustine church, Siena, Italy) were made of a variety of materials, including chalk white, leaden white, yellow ochre, red ochre, red lead, cinnabar, Siena earth, green earth, verdigris, azurite, and carbon black^19^.

A polychrome plasterwork stucco (zigzag arches in the Hall, Salón de Embajadores, Mudéjar Palace of the Real Alcázar of Seville, Spain) was studied using XRF, XRD, and FTIR. It was discovered to be made up of 1–3 microlayers with a thickness of 5 and 300 μm, composed of blue (19.88% Cu-azurite), vermilion (19.00% lead Pb-Mascotte and 5.34% cinnabar Hg), and gilded layers (68.93% Au)^20^. In order to examine the 15th and 16th-century vault paintings in the Cathedral of Our Lady (Antwerp, Belgium), XRF and Raman spectroscopy were used. The presence of the strong Raman scatterer calcite made it difficult to identify the paints by Raman spectroscopy, while the presence of gypsum on the mediaeval vault painting may indicate deterioration^21,22^.

The Bahri Mamluks architecture of Sultan Hassan's complex features a novelty in that all of the components originate from one larger block, which is the block of the main building^23^. Since then, it has been refined to the point where it is now widely acknowledged as evidence of the splendor of Mamluk architecture. Due to the extensive trade with the Catalans, Alexandria and Barcelona were able to reestablish communication during the reign of Sultan Qaytbay, which is what is responsible for the emergence of Andalusian design characteristics^12^. Why the components present here were absent from earlier structures is unclear. Due to the favorable connections with Catalans and the free flow of people and products over the Mediterranean, there are numerous similarities to the reign of al-Nasir Mohamed^12^.

Fungi have the ability to colonize and degrade a variety of inorganic and organic artifacts made of materials including wood, textile, historical paper, stones, albumen silver prints and stucco that date to all periods of civilization^24–27^. In humans, *Aspergillus* spp. can result in various chronic infections that are prolonged and noninvasive forms. This disease is characterized by diverse symptoms, so follow-up should be prompt and serious to counteract this fungus ^28^. Conidia are produced in chains on separate phialides that emerge from distinct conidiophores^29–31^ and formation of ascospores from sexual reproduction^32^. As part of cultural heritage objects, *Aspergillus niger*, *A. flavus*, and *A. terreus* were isolated from the biodeterioration of archaeological manuscripts, gypsum board antique, archaeological fabric^33^. All archaeological materials including cartonnage, limestones, sandstones, and wood stuff, are capable of being colonized by *A. niger*, which consumes major elements and structures^34–40^. These fungi produce enzymes in their hyphae that can break down organic materials into tiny molecules that the fungus can consume as nutrients^41^.

1,4-*β*-glucosidases, exo-1,4-*β*-glucanase, and endo-1,4-*β*-glucanases are xylanases and hydrolytic enzymes which produced by *Aspergillus* spp. for the hydrolysis of cellulose as well as endo-1,4-*β*-xylanases and *β*-xylosidases for the hydrolysis of hemicelluloses^42,43^. Glucoamylase, xylanases, and arabinases are released in the *A. niger* mycelium peripheral to break down hemicellulose^44,45^, whereas xylan, a type of hemicellulose, can also be degraded in the presence of *A. niger* ^46^. Enzymes, cellulase and xylanase, produced by *A. niger* are able to degrade the waste products from growing forestry tress including *Pinus roxburghii*, *Toona ciliate*, *Cedrus deodara*, and *Celtris australis*^47^.

In the present work, the exquisite decoration of the wooden panel in Qaytbay’s mosque was covered with gypsum, then painted with blue with a gilded layer. Due to environmental variables that cause biological degradation, this area of Cairo is vulnerable to major indicators of deterioration. Due to numerous reasons, including bird droppings, insects, and fungal growth, the wooden panel of Qaitbay was severely damaged. The effects of *Aspergillus* fungi on the wooden panel (pine wood- stucco, painting), were the main focus of this investigation.

In order to ascertain the characteristics and chemical composition of the painted and gilded stucco over the wooden support, the current work aims to implement a multi-scale characterization to assess and examine the painted and gilded wooden panel with stucco decorations. This study also aims to determine the effects of *Aspergillosis* fungi that are present on the surface of the wooden panel exist on the surface of the wooden panel in order to create a suitable preservation and restoration plan in the future after putting the experimental study into practice based on the findings of the examination and analysis, which had a significant impact on the historical Mosque and the well-being of the worshipers.

## Materials and methods

### Visual observation

**Figure 1:**
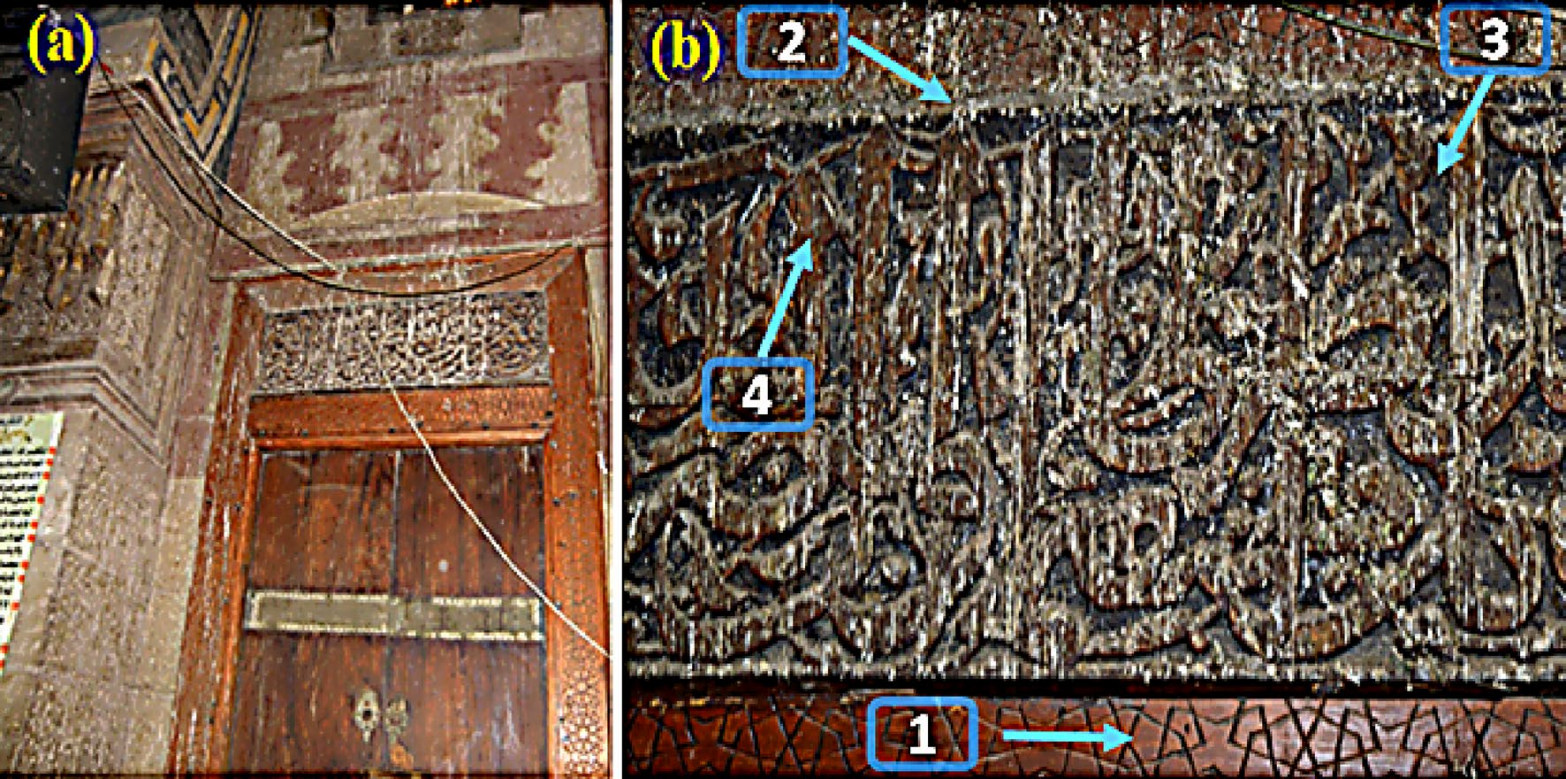
Showing an inscription-supported wooden panel covered with a layer gilded and the painted layer above a wooden door, Qibla wall, Sultan al-Ashraf Qaytbay, Egypt (**a**); Details of the archaeological decorative unit (**b**) consists of (1) wooden support, (2) stucco layer, (3) blue paint layer (Azurite), and (4) gilded layer (Photos were taken by co-author ALyaa G. A. I. Hassan).

This study complied with relevant institutional, national, and international guidelines and legislation. This study does not contain any studies with human participants or animals performed by any of the authors. An inscribed wooden panel covered with a layer of painted and gilded stucco above the wooden door in the qibla wall from the south side leading to the dome of the mausoleum of Sultan al-Ashraf Qaytbay, Cairo, Egypt. The wooden panel used in this complex has a stucco layer composed of calcite with traces of gypsum on top, followed by a final layer with a blue paint background and inscriptions in of Arabic letters contains a Quranic verse from the beginning of Surat Al-Fath, written in the technique of gilding (Fig. 1a, b). The panel is 140 cm long, 40 cm wide, and 32 cm thick; the thickness here refers to the depth of the panel from the outside part to the wall level.

**Figure 2:**
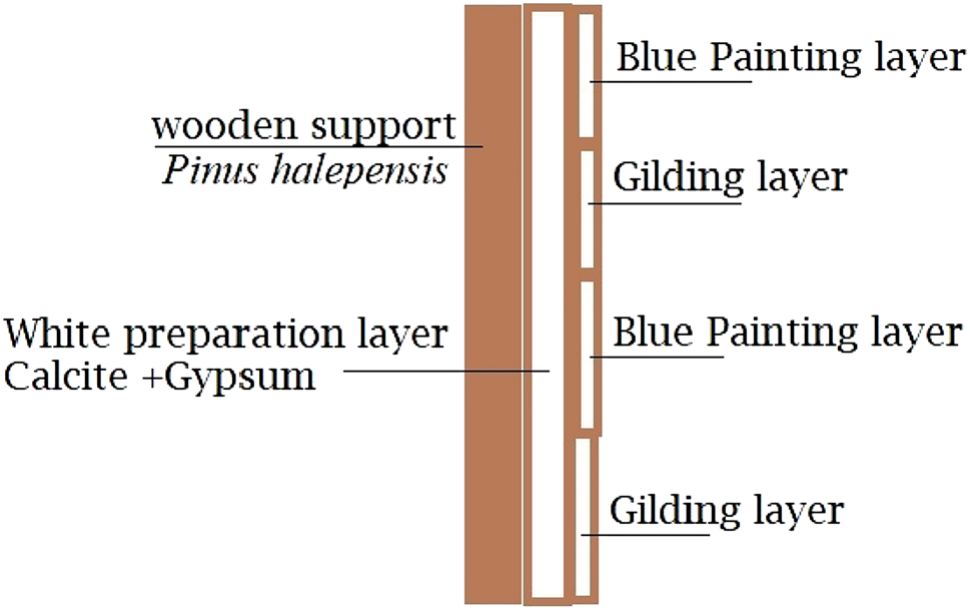
Graph illustrate the stratigraphic composition of the wooden panel case study, (materials identification added after examination and analysis results).

The most common method of evaluation was with the unaided eye, and with careful inspection, skilled conservators were often able to determine the materials of the objects, the color scheme employed in the painted layers, and some noticeable deterioration features. By visual inspection, we can identify the panel's foundation material, which is a wooden support coated in a priming layer (white color), followed by a blue glaze and inscriptions with a gilded layer over the primer layer (Fig. 2).

The visually observed considerable coating deterioration in the damage wood support may have been caused by a fungal infection^48–50^. There was also damage to the gilding layer, as well as the disintegration of the white ground, paint layers, and layers of paint. It is well-recognized that fungi may grow on organic and inorganic materials, causing them to change and decompose^51^.

### Analytical and survey study

To avoid causing any harm to the panel, the samples for the analysis of the piece under study were taken from the edge of the panel, from the white priming layer, in a hidden portion. Fallen pieces of the artifact served as analytical samples for the blue-painted and gilded layers.

#### Identification of a wood sample by microscopic examination

An optical microscope equipped with digital camera at X80, and a stereo microscope at X120 were utilized to examine the transverse and longitudinal sections of wood. To identify the main anatomical features of the wood, images were captured using a scanning electron microscope (SEM) (JEOL 6400 SEM attached with EDX unit combined system energy dispersive spectrometer magnifications 500× and 200×. To identify the species of wood, high-magnification SEM images (200 µm) of the cross-sectional wood structure were examined. Additionally, The SEM instrument was used to identify the different thicknesses of each layer of the painted stucco supported on a wooden panel.

#### The Elemental composition of the priming layer

The elemental composition of the priming layer, as well as the blue and gold-coated layers were analyzed by the Energy Dispersive X-ray analysis (EDX) technique. The analysis was done for preparation layer at six points, two points for the gilded layer, and one point for the blue paint analysis.

#### X-ray diffraction analysis (XRD)

The chemical composition of painted and gilded stucco material as a ground layer applied on the wooden support, was characterized using X-ray Diffractometer—High Resolution (PANalytical X'Pert PRO MRD) equipment model X’Pert PRO with Monochromator, Cu-radiation (ƛ = 1.542 Å) at 45 kV and 35 mA.; the scanning speed 0.03°/s^52^. The reflection peaks between 2ϴ = 2° and 60°, the corresponding spacing (d, Å) and relative intensities (I/Io) were obtained. diffraction and relative intensity plots were obtained and compared with ICDD files.

#### Fourier transform infrared spectroscopy—attenuated total reflectance (FT–IR–ATR) analysis

Fragment samples were analyzed to determine the type of organic medium used the white preparation layer coating by Fourier transform infrared spectroscopy—attenuated total reflectance (FT–IR–ATR), using a Bruker Vertex 70 FT–IR spectrometer equipped with a detector using crystal ATR, which represents is additional scans at 2 mm/s in a spectral region ranging from 4000 to 500 cm^−1^, with a resolution of 4 cm^−1^^53^.

### Isolation of fungi

#### Culturing of fungi

Aspergillosis fungi were isolated from all layers of the wooden panel structure of Qaitbay's Mosque (wooden support, gypsum, blue paint, and the gilded layer) as shown previously in Fig. 1 fungi were isolated by rubbing with swabs on different culture media. Three growing media were used to isolate the fungi; (1) Potato dextrose agar (PDA), (Potato 200 g, Dextrose 20.0 g, Agar 15.0 and distilled water 1000 mL, with final pH: 5.6 ± 0.2), (2) Sabouraud Dextrose Agar (SDA) (Dextrose 40 g, Peptone 10 g, Agar 15 g and 1000 ml distilled water and (3) Czapek Solution Agar (Sucrose 30.0 g, Sodium Nitrate 2.0 g, Dipotassium Phosphate 1.0 g, Magnesium Sulfate 0.5 g, Potassium Chloride 0.5 g, Ferrous Sulfate 0.01 g and Agar 15.0 g)^54–57^. All the media components were autoclaved at 121 °C for 20 min. Fungi have been identified based on their taxonomic keys^58,59^.

#### DNA isolation, PCR amplification and sequencing

The total genomic DNA was extracted from the three molds using Norgen’s Plant/Fungi DNA Isolation Kit (Product No. E5038, Merck KGaA, Darmstadt, Germany) following the manufacturer's instructions. The fungal ITS region was amplified using the primers ITS1 (5′-TCCGTAGGTGAACCTGCGG-3′) and ITS4 (5′-ATCCTCCGCTTATTGATATGC-3′)^60^. The PCR mixtures were as follows: 10 μl of 2 × master mix, 1 μl of each primer (10 μM), 1 μl of template DNA and 13 μl of H_2_O. PCR conditions consisted of an initial denaturation at 95 °C for 4 min, followed by 35 cycles at 95 °C, 52 °C and 72 °C each for 30 s, and a final elongation at 72 °C for 7 min. PCR products were purified and sent for sequencing at Macrogen Co. in Seoul, Korea. The sequences after that were deposited in Genbank under accession numbers.

### Experimental models

#### Preparing and collecting mimic samples

In order to better mimic the original material, The wood samples were appropriately worked to have dimensions of 2 × 2 cm and a thickness of 1 cm. In order to create the animal glue solution, which is used to bind the particles of the primer layer and paints as well, animal glue beads were utilized. Animal glue is made by soaking in a hot water bath with a temperature not exceeding ~ 60 °C until the pure gelatin swells up with the water.

The ground layer employed in our object is composed of calcium carbonates (calcite) and hydrated calcium sulfate (gypsum), according to the EDX elemental analysis. These were the same components used to implement the ground coating during the eras of ancient Egypt^61,62^. Therefore, the ground layer was prepared by mixing fine white calcite and traces of gypsum with water and animal glue binding media. The mixture was then applied to the wooden samples and allowed to dry for 48 h at the room temperature, ensuring that the coating was entirely dry.

**Figure 3:**
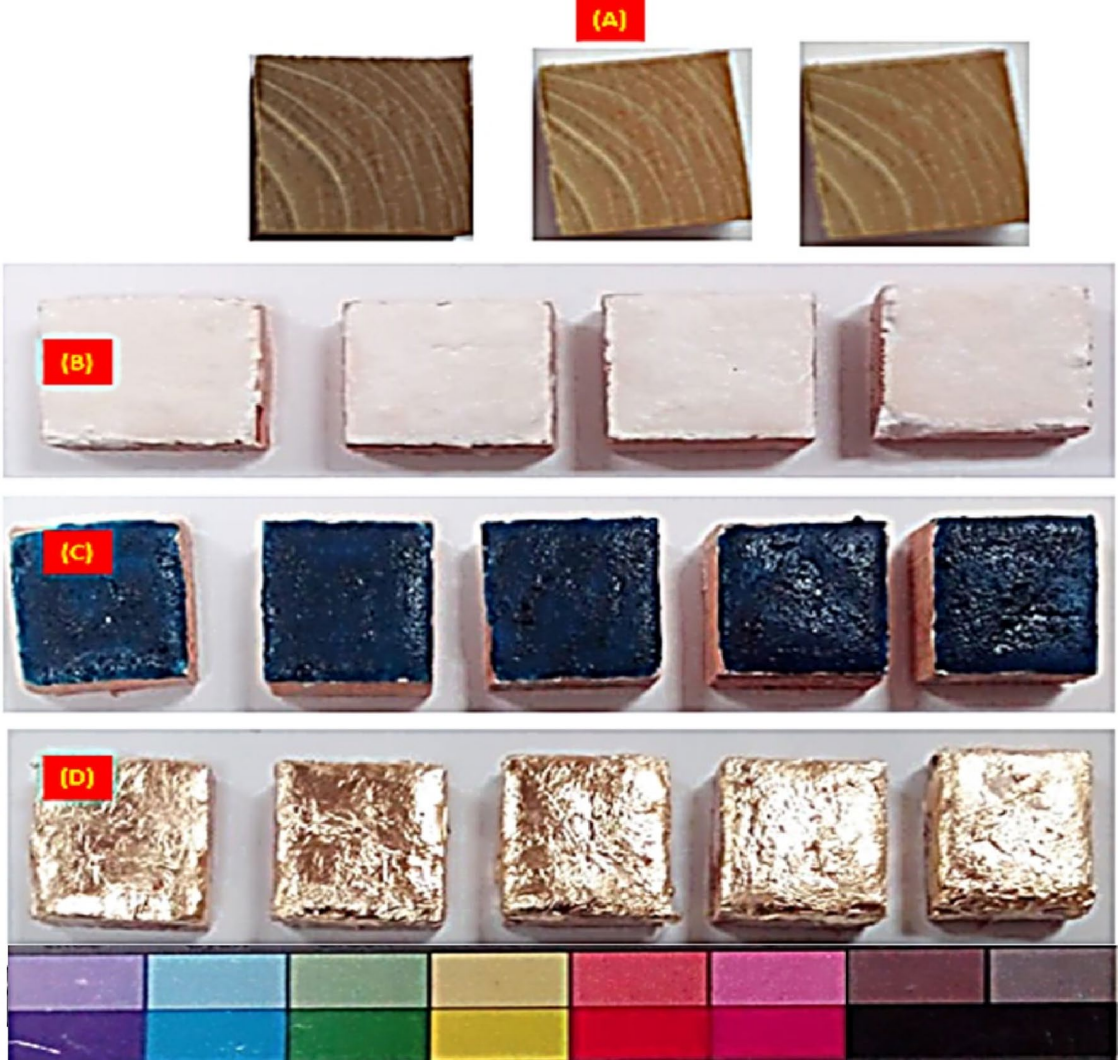
The created four groups of experimental samples: (**A**) Pinewood specimens that are uncoated. (**B**) Wooden samples with white ground layer (made of calcite and gypsum); (**C**) Wooden samples with white ground layer and blue paint layer (azurite paint); (**D**) Wooden samples with white ground layer and a gilding layer.

Similar to the original paintings, natural paint was utilized for the piece. The paint used is #10206 Blue (Azurite) blue lacquer produced by Kremer Pigments Inc. The animal glue binding media and powder paint are combined immediately before being applied over the white primer and allowed to dry for roughly seven days (Fig. 3).

The limestone powder and traces of gypsum that were used to form the ground layer applied to the wooden specimens were coated with gilded paper, which was then dried for 48 h to make ensure it was completely dry. A historical investigation has looked at similar recipes and material for the same period as well as earlier times to prepare replicas^2,17,63,64^.

We can divide the samples into four groups using the earlier techniques for creating experimental samples as follow:

***Group A***: wooden samples (Pinewood) without any coatings.

***Group B***: wooden samples covered with a white ground layer (calcite + gypsum).

***Group C***: wooden samples covered with a white ground layer then covered with a blue painted layer with Azurite paint.

***Group D***: wooden samples covered a with white ground layer then covered with a gilt layer.

#### Fungal colonization test

The prepared four groups from the typical experiment were inoculated separately, with each fungus tested under the laboratory conditions. Each fungus was given a suspension of spores, to which 10 ml of sterile distilled water was added to fungal culture dishes containing PDA medium (7-day-old), and spores were spread using a camel hairbrush. Using a hemocytometer slide, spore suspensions were then individually filtered through gauze with a standard concentration 1.2 × 10^6^ spores/mL^27,65,66^. After six months, colonization was assessed in both standard and inoculated samples to illustrate the three fungi's growth and degradation patterns using SEM examination^31^.

### Color Change in the CIELAB System

In our case, the chromatic change of the mimic experimental samples is used as a tool to evaluate the effect of the fungal growth on the samples. The colorimetric measurements taken into consideration are the main tool used to evaluate the color change (ΔE) due to the effect of deterioration or changing fungal or environmental conditions.

ΔE was performed in the color space CIE L*a*b*, where L denotes black-to-white color, a :green-to-red color, and b :blue-to-yellow color. The delta values of the parameters (∆L*, ∆a* and ∆b*) and the total color changes (ΔE) for the samples were measured using the following formula ^48,67,68^;

$$\Delta E = \sqrt {\left( {\Delta {\varvec{L}}} \right)^{2} + \left( {\Delta {\varvec{a}}} \right)^{2} + \left( {\Delta {\varvec{b}}} \right)^{2}}$$

where = (∆L)^2^, (∆a)^2^, and (∆b)^2^ are the differences between the values of the color indices before and after fungi treatment.

## Results and discussion

### Analytical and investigation study of the archaeological wooden panel

#### Identification of wood sample by light microscope, stereo microscope and Scanning Electron Microscope (SEM)

**Figure 4:**
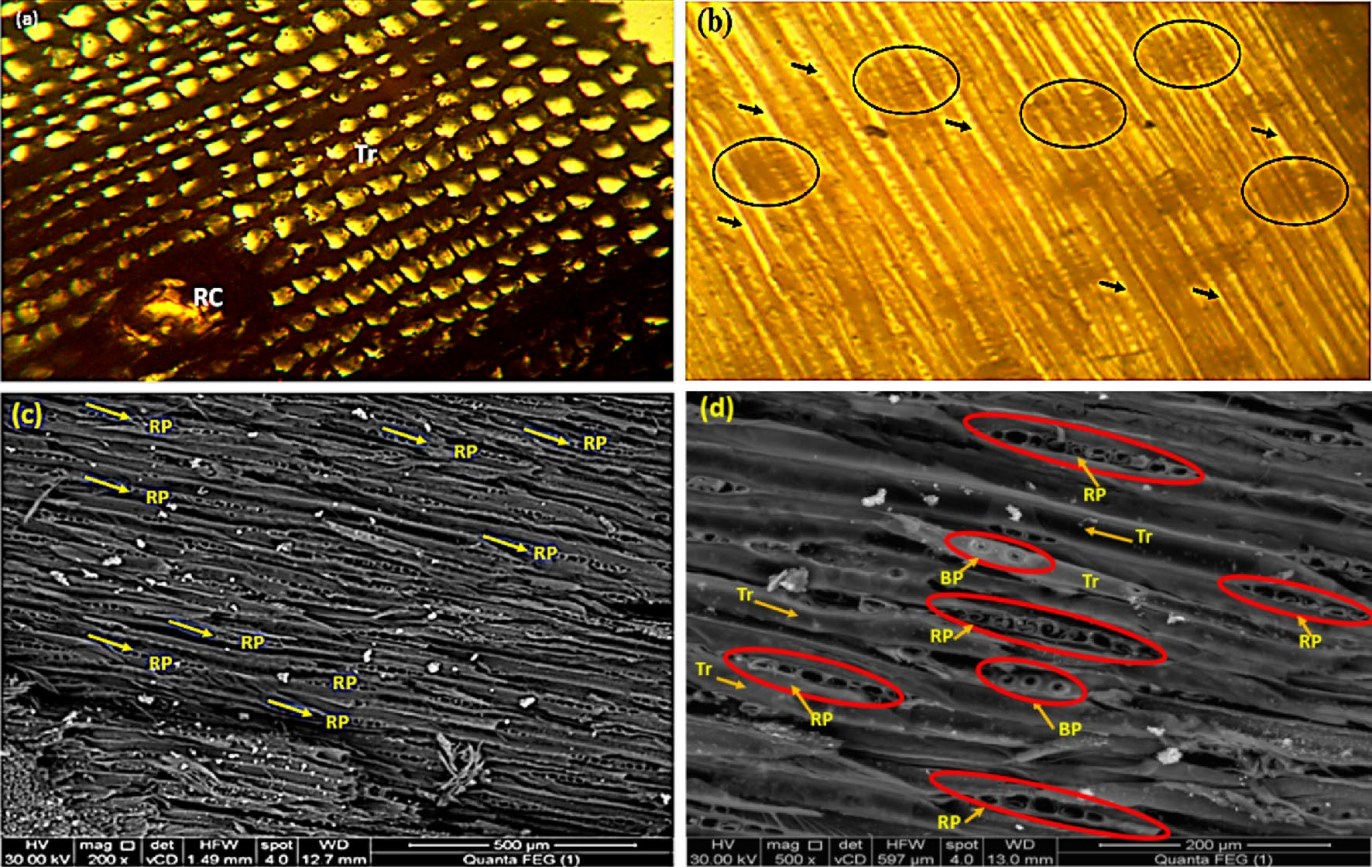
Identification of wood species. (**a**) A light microscope for a transverse section; (**b**) A stereo microscope displaying a longitudinal section with tracheids (arrows) and the rays (circles); SEM images of the tangential section of the pinewood panel taken at two different magnifications, (**c**) at 500 µm and (**d**) at 200 µm. RP: Uniseriate-type ray parenchyma; BP: Bordered pits; Tr: Tracheids, and RC: Resin canal.

To identify the type of wood used, two incredibly tiny samples were extracted from the written tape and examined under a light and stereo microscopes. The two specimens from the written tape appeared to be made of pine wood in cross section (Fig. 4a) under a light microscope and longitudinal section (Fig. 4b) under a stereo microscope, where tracheids, resin canal, and the rays were identified.

The wood sample was recognized as pinewood by scanning electron microscope (SEM) images acquired in cross-section 500× and 200× magnifications (Fig. 4c,d). The wood structure demonstrated that the wood sample is a pinewood (Fig. 4c,d). Uniseriate rays and bordered pits appearance in the cell wall of the tracheid define the wood. In this regard, the wood is related to the Aleppo pine (*Pinus halepensis*), which was previously found in the wooden ceiling of the Madrasa of Al-Ashraf Qaytbay in Cairo, Egypt^2^.

#### Examination of the wooden panel layers

**Figure 5:**
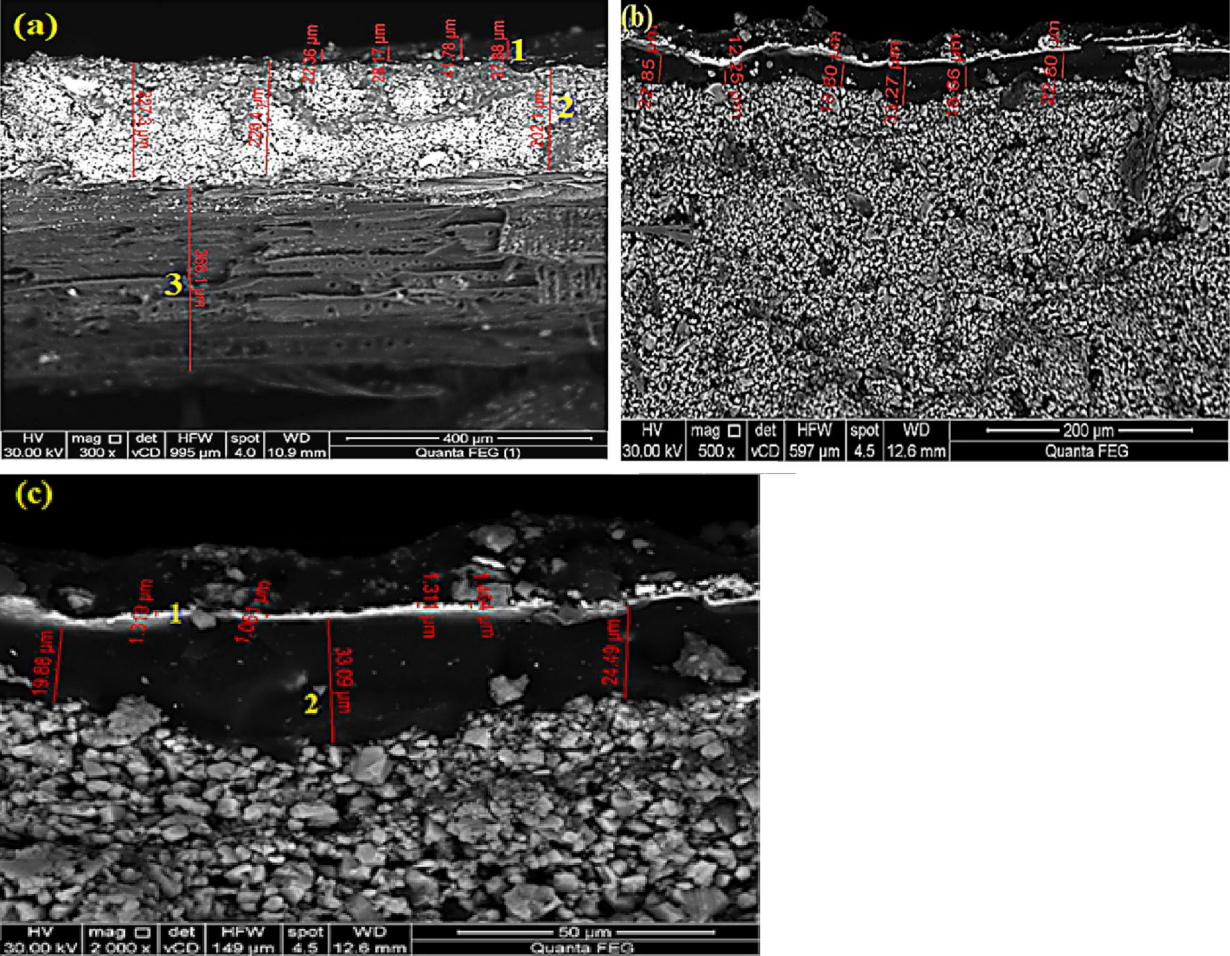
SEM images demonstrating the various layer thicknesses of painted stucco that is supported by a wooden panel, (**a**) gild or Azurite paint layer at point 1, preparation layer at point 2, and wooden support at point 3; (**b**) blue paint layer; and (**c**) gilding layer at point 1 and blue paint layer at point 2. Lines indicate the layer thickness.

The wooden panel has three layers that are visible at a magnification of 400 µm: a layer of pinewood backing that is 368.1 µm thick, a priming layer that is 228.11 µm thick, and a layer of gild or Azurite paint that is 32.11 µm thick (Fig. 5a). Additionally, blue coating (Azurite paint) with a thickness of 19.60 µm can be anticipated from Fig. 5b can be expected. The gilding layer was 1.03 µm thick and the azurite layer was 25.82 µm, according to Fig. 5c.

These findings supported previous research, such as measurements and characterizations of Mamluk coating materials in Cairo, Egypt: El-Ashraf Bersbay Madrasa (826 A.H/1423 A.D) stated that the wooden panels were covered with a layer of calcium carbonate, chalk, and bonded with animal glue ^63^. The calcite material layer was coated with white lead, which was then hidden by a layer of gilded bole. Due to the potential that it has worn over the original gilded layer paper, the ceiling above the shaft has also undergone re-gilding^17^.

Similarly, to achieve a pleasing look, cartonnages were covered in a layer of gypsum mixed with an organic binding medium made of animal glue and natural mineral oxide^69^. King John I’s sword was made of silver in the nineteenth century (coffer 27), and crowns, belts, scepters, swords, and rosary beads were all decorated with gold paint^17^.

As consolidates and adhesives for organic and inorganic materials, animal glues are natural polymers derived from fish collagen, connective tissue, cartilage, and bones^70^. Gilded tin foil in the fresco was used by medieval painters to decorate the corona with noticeable fungal spores where lead-based paints were applied ^19^.

#### Elemental analysis by EDX

The initial composition of the stucco ground layer analyzed by EDX (Table 1) of six points showed the presence of C 50.93%, O 33.82%, Ca 11.45% and Si 6.46% in the first point (Fig. S1a), O 32.33%, C 27.76%, N 7.61%, Na 4.72%, Ca 7.65%, Si 6.59%, and Cl 4.85% in the second point (Fig. S1b), C 62.02%, O 28.86%, Si 2.19% and Ca 2.28%, in the third point (Fig. S1c), C 35.21%, O 29.66%, Si 15.39%, Mg 4.61%, Al 3.67%, K 3.53%, S 3.25% and Ca 2.91% in the fourth point (Fig. S1d), C 36.80%, O 36.80%, N 10.93%, Ca 4.32% and Si 3.69% in the fifth point (Fig. S1e) and C 51.18%, O 29.53%, Ca 3.88%, Fe 3.20%, Si 3.20%, and S 2.71% in the sixth point (Fig. S1f) major elements with other minor elements. as initial composition indicated that the ground layer was made from gypsum.

**Table 1:** EDX elemental composition of the ground layer from six points.

Element	Point a		Point b		Point c		Point d		Point e		Point f	
	Wt%	At%	Wt%	At%	Wt%	At%	Wt%	At%	Wt%	At%	Wt%	At%
C	36.59	50.93	27.76	39.19	48.85	62.02	21.33	35.21	26.00	36.80	33.88	51.18
N	nd	nd	7.61	9.22	nd	nd	nd	nd	9.01	10.93	nd	nd
O	32.37	33.82	32.33	34.26	30.27	28.86	23.93	29.66	34.65	36.80	26.03	29.53
Na	1.23	0.89	4.72	3.48	1.17	0.77	0.80	0.69	1.50	1.11	2.34	1.85
Mg	0.90	0.62	1.01	0.70	0.36	0.23	5.65	4.61	1.40	0.98	0.45	0.34
Al	2.38	1.48	1.53	0.96	1.57	0.89	4.99	3.67	2.04	1.28	1.35	0.90
Si	6.46	3.84	6.59	3.98	4.04	2.19	21.80	15.39	6.09	3.69	4.95	3.20
P	nd	nd	1.25	0.69	0.44	0.22	nd	nd	1.45	0.80	0.63	0.37
S	2.75	1.43	1.42	0.75	1.38	0.66	5.25	3.25	1.57	0.83	4.79	2.71
Cl	1.91	0.90	4.85	2.32	1.32	0.57	1.92	1.07	1.74	0.84	2.69	1.37
K	0.94	0.40	1.72	0.74	0.86	0.33	3.53	1.79	2.34	1.02	0.87	0.40
Ca	11.45	4.78	7.65	3.24	6.00	2.28	5.89	2.91	10.20	4.32	8.57	3.88
Ti	nd	nd	nd	nd	0.46	0.15	nd	nd	nd	nd	0.51	0.19
Fe	3.03	0.91	1.56	0.47	1.78	0.48	4.90	1.74	2.01	0.61	9.85	3.20
Cu	nd	nd	nd	nd	0.73	0.17	nd	nd	nd	nd	1.16	0.33
Zn	nd	nd	nd	nd	0.78	0.18	nd	nd	nd	nd	1.92	0.53

nd: not detected; Point a: Stucco sample from the far right of the writing tape; Point b: Stucco sample from the far left of the writing tape; Point c: Stucco sample from the middle of the written tape; Point d: From the far right of writing; Point e: Stucco sample from the middle of the writing; Point f: Stucco sample from the far left of the writing.

The elemental compositions of the blue coating layer, as determined by EDX analysis (Table 2) were Cu 47.18%, C 25.31%, Ca 11.64%, Zn 6.72%, O 5.50%, Si 1.84%, and Fe 1.82%, which suggests that the blue coating of the panel was made of Azurite [(Cu_3_(CO_3_)_2_(OH)_2_)] (Fig. S1).

**Table 2:** EDX elemental composition of blue paint layer.

Element	Wt%	At%
C	25.31	57.19
O	5.50	9.33
Si	1.84	1.78
Ca	11.64	7.88
Fe	1.82	0.88
Cu	47.18	20.15
Zn	6.72	2.79

According to the EDX analysis of two points of the gilded layer (Table 3),the first point's original compositions were O 39.84%, C 25.03%, Ca 10.81%, Au 8.64%, Cl 5.89%, and Pb 4.20%(Fig. S1a), while the second point's compositions were O 35.12%, C 21.55%, Ca 18.16%, and Au 17.94% (Fig. S1b). The presence of Pb (lead), however, may imply the use of an alloy of gold and lead to give better durability, although this has not been proved because the amount of lead is quite little. These compositions of the key elements show that the gilded layer employed was mostly formed of gold. Because of its symbolic nature, gold has been utilized extensively since ancient Egypt and even now to gild funerary objects^8^.

**Table 3:** EDX elemental composition of gilded layer.

Element	Point a		Point b	
	Wt%	At%	Wt%	At%
C	6.92	25.03	4.86	21.55
O	14.68	39.84	10.56	35.12
Na	nd	nd	0.44	1.02
Pb	20.04	4.20	nd	nd
Al	0.63	1.02	0.74	1.46
Si	1.61	2.49	1.16	2.19
K	1.30	1.44	1.32	1.80
Cl	4.81	5.89	nd	nd
Au	39.20	8.64	66.43	17.94
Ca	9.98	10.81	13.68	18.16
Fe	0.82	0.64	0.80	0.76

nd: Not detected; Point a: From the middle of gilding layer; Point b: from the edge of gilding layer.

The composition is pure gold with traces of other elements, according to the aforementioned elemental analysis by EDX, but the highest ratio of elements after gold (Au), is Ca, which makes sense given that the ground layer was primarily composed of calcium carbonates with a trace quantity of gypsum. Additionally, there is Pb, particularly in point 1, which, according to the results, can be added to gold to improve its properties, though we are unable to confirm this due to the low ratio and unusual use of it with gold for alloys, particularly during the Islamic era for ceiling and building decorations. It might have happened as a result of erosion, or traces of the ground layer. For instance, although gold-plated surfaces may appear solid and metallic, they are actually very delicate and prone to damage. The majority of gilded paper is extremely soft and can be easily faded or discernible if not handled carefully^71^. Unfortunately, we lack sufficient information regarding its composition and method of application.

Gilding, which is the practice of applying gold to a surface for decorative purposes, has long held a significant place in Islamic civilization’s decorative arts, particularly the gilded ceilings^71^. Since gold foil has a medium thickness compared gold sheets, it was probably used to create the gilded layer rather than gold sheets. During the Islamic era, the gilding technique was an important and popular approach for decorating surfaces and a variety of arts^72^.

#### X-ray diffraction analysis (XRD)

XRD spectra of the deep ground layers of the wooden panel with its ground coating layer (Table 4) from several points demonstrated the presence of gypsum at 3–10% and calcite at 75–90% with a minor quantity of quartz at 2–5% (Fig. S1a–d) in addition to 10% crystalline cellulose.

**Table 4:** Chemical percentage of the wooden panel and ground layer by XRD analysis.

Ref. code	Chemical name	Chemical formula	Semi*-*quantitative (%)			
			Point a	Point b (ground layer only)	Point c	Point d
01-072-4582 01-083-0578 01-076-2713 01-072-1652	Calcite	CaCO_3_	90	95	75	80
00-002-0471 00-003-0444	Quartz	SiO_2_	nd	2	5	nd
00-050-2241 00-050-2241 00-050-2241	Cellulose	(C_6_H_10_O_5_)n	10	nd	10	10
01-074-1904 00-006-0047 01-074-1904	Gypsum	CaSO_4_·2H_2_O	nd	3	10	10

nd: Not detected.

These findings are in line with research on ancient artifacts from Egypt or other nations. Because of its symbolic meaning of, gold often employed, especially when adorning burial artifacts^8^. Gypsum and calcite were previously found to be the key ingredients of the initial layer of the golden layer in the ceiling of the Qaitbay madrassa, with evidence of Hematite^2^. Calcite and quartz were utilized in the ground layer of a mural in the Ain el-Lebekha Temple located in Kharga Oasis, New Valley Governorate, Egypt ^64^.

Polychrome stucco paints found in historical buildings in Spain contained mineral paints such as azurite, gilt-coat layer-leaf, and vermilion-litharge according to XFR, XRD, and FTIR analyses^20,38^. Renaissance vault paintings from the fifteenth and sixteenth centuries were examined using XRF, which turned up paints such as red lead, hematite, lead white and azurite^21^. Three Ilkhanid monuments, the madrasas of Sayed Shams-al-Din, Sayed Kamal-al-Din, and Sayed Rukn-al-Din in Yazd, Iran, were found to have an azurite layer (blue coating layer), which was identified by XRD analysis and employed as a blue-colored copper carbonate^73^. It is abundantly obvious that azurite, either in its pure form or combined with other blue paints such as papagonite, was used as a blue coating in the decoration of ceilings of Islamic buildings from the Fatimid era until the Ottoman era^74^.

The facts and sources mentioned above lend credence to the idea that azurite is present, and even if Si makes only a small portion of the sample, it can be traced back to the white primary layer.

#### Fourier transform infrared spectroscopy (FTIR) analysis

**Figure 6:**
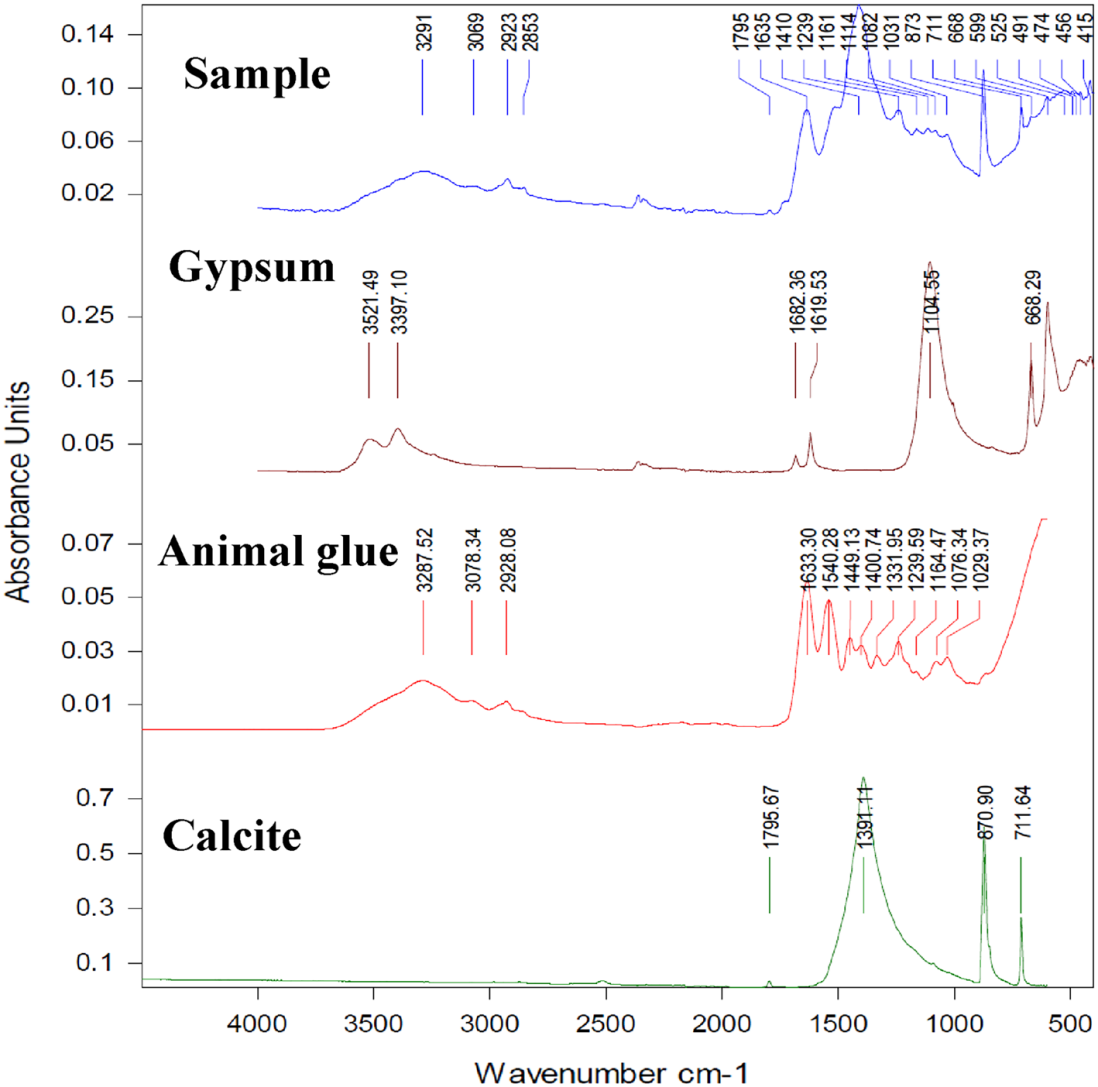
FTIR–ATR spectra of the ancient sample, animal glue, calcite, and gypsum.

The FTIR spectrum revealed that animal glue was used with gypsum and calcite to perform preparation or ground painting layer (Fig. 6 and Table 5). According to http://www.irug.org/search-spectral-database?reset=Reset, the characteristic bands of animal glue are amide I (peptide carbonyl group C=O stretched at ≈1646 cm^−1^), amide II (C-N stretched together with the N–H in-plane bending at 1535 cm^−1^), and amide III (N–H bending, CN stretching vibration, and small contributions from both the in-plane CO bending and CC stretching vibration at 1236 cm^−1^). The characteristic bands of calcium carbonate appear at 1412 cm^−1^ (CO_3_^–2^ stretching band) and 875 cm^−1^ (O–C–O curvature of the carbonate group). The characteristic bands for gypsum are asymmetric for SO_4_^–2^ and stretch at 1138 cm^−1^, and the S=O stretches for SO_4_^–2^ at 1618 cm^−1^ and 1680 cm^−1^. The FTIR spectra contain the superimposed spectra of each component present since materials containing mixes, like in our instance, frequently do. Each component's concentration is directly inversely correlated with the intensity of its bands^75^.

**Table 5:** FTIR of functional groups of ancient and animal glue standard specimens.

Wavenumbers (cm^−1^)	Functional groups and comment
3610.7 3596.7	Asymmetric and symmetric O–H stretching bands of both animal glue and gypsum (splitting of the band is due to high concentration of gypsum in the sample). N–H stretching band of animal glue is overlapped by O–H bands
2993.6 2885.5	Asymmetric and symmetric C-H stretching bands of aliphatic groups of animal glue
1681 1627.9	Region of carbonyl stretching band (amide I) of animal glue. Splitting occurs due to S=O of SO_4_^–2^ of gypsum
1512.2	Combination of C-N stretching band and N–H bending band (amide II) of animal glue
1473.6	Broad band due to combination between C-H bending vibration of animal glue and CO_3_^–2^ stretching band of calcite
1157.2	C–O stretching of animal glue + Asymmetric SO_4_^–2^ stretching band of gypsum
879.5	O–C–O bending band of carbonate group of calcite
663.5 609.5	SO_4_^–2^ bending band of gypsum

The resulting spectrum may be complex, with overlapping bands obscuring those required to identify individual substances. The other possibility was the effect of surrounding environmental damaging factors, particularly high temperatures, which moved the functional groups until they completely disappeared, resulting in mechanical property breakdown and loss of binding media function.

The FTIR spectrum reveals the presence of animal glue with gypsum in the preparation layer Because animal glue was frequently employed for coatings applied to wooden panels, the FTIR spectrum reveals the presence of animal glue along with gypsum in the preparation layer This outcome is in line with other binding materials utilized in ancient Egypt^76^. A fresco from the years 1773–1774 and a painted wood panel, both obtained from the cross-sections of model samples and historical paintings, have been found to contain animal glue from the Renaissance period^77^. Animal glue was detected using FTIR on samples taken fine layers of artwork from various periods (16th to eighteenth centuries)^78^. SEM–EDX and laser-induced breakdown spectroscopy (LIBS) analyses of sycamore wood, which was commonly used in ancient Egyptian civilization, revealed that it had been covered with preparation layers of calcium carbonate and mixed with prepared animal glue^79^. Five Russian icons from 16th to eighteenth centuries were authenticated using FTIR and XRD investigations, which revealed that the ground white preparation layers of gypsum covering the wooden support had a variable thickness (0.2–0.4 mm)^80^.

### Identification of isolated fungi by molecular characterization

In order to accurately identify the three molds obtained in the present study, we extracted the DNA, amplified the fungal-ITS region, and sequenced the purified amplicons. The obtained sequences proved the initial identification process. Our fungal isolates were confirmed to be *Aspergillus niger*, *A. flavus*, and *A. terreus* according to the alignment of the obtained sequences at the NCBI portal, and the depositing accession numbers were OQ820164, OQ820163, and OQ820160, respectively.

The *Aspergillus* genera are the most found fungi that cause the most deterioration to the object, e.g., *A. niger*, *A. flavus*, *A. glaucus*, *Trichoderma album*, *T. glucum*, *T. koningi*, *Penicillium chermesimum*, *Alternaria alternata*, and *Fusarium nivale* were previously isolated from weathered decorated wood of the painted ceilings of Qaitbay's madrassa, Egypt^2^. Dangerous Aspergillosis spreads widely spread in Egypt and its effects of human and inorganic materials^81^. Because the mosque is opened five times a day for prayer and provides the right temperature and humidity for these species of fungi, *Aspergillus* species are common there. Using an Andersen air sampler for a year, the prevalence of airborne mold spores was assessed at four locations in Cairo. Seasonal variations in the average total count of molds for *Aspergillus* rot were recorded (27.2%)^82^.

### Evaluation of fungal infestation on mimic samples

#### Color change

The color change (ΔE) of ground-coated and gild-coated samples as a result of *A. niger*, *A. flavus* and *A. terreus* colonization is shown in Table 6. ΔE values for *A. niger*-colonized wood + gypsum + gilded layer, wood + gypsum, wood + gypsum + blue painted layer and pinewood without coatings were recorded as 33.37, 30.06, 26.60 and 13.71, respectively. *A. niger* had the highest ΔE value (52.29) in wood + gypsum + gilded layer colonized with *A. flavus* followed by wood + gypsum (30.39), wood + gypsum + blue painted layer (ΔE 24.41) and pinewood without coating, (16.67). For the effect of *A. terreus*, the highest ΔE (44.12) was recorded with the model samples wood + gypsum followed by wood + gypsum + gilded layer (42.05), wood + gypsum + blue paint layer (22.33) and pinewood without coatings (14.17). Therefore, the highest ΔE fount in wood + gypsum, wood + gypsum + blue paint layer, and wood + gypsum + gilded layer was reported by the colonization with *A. terreus*, *A. flavus* and *A. flavus*, respectively, when compared to controls (wood without coatings).

**Table 6:** The chromatic parameters measured for the samples in the L*a*b* (CIE 1976) color system.

Material		*Aspergillus niger*				*Aspergillius flavus*				*Aspergillius terreus*			
		L*	a*	b*	ΔE	L*	a*	b*	ΔE	L*	a*	b*	ΔE
Pinewood without coatings	S*	26.85	14.01	55.13	–	26.85	14.01	55.13		26.85	14.01	55.13	–
	E	41.56	12.17	27.51	13.71	40.92	7.63	20.89	16.67	41.89	10.22	20.89	14.17
Wood + Gypsum	S*	98.89	5.52	5.45	–	98.89	5.52	5.45	–	98.89	5.52	5.45	–
	E	71.82	4.43	18.57	30.06	70.63	4.63	16.69	30.39	56.10	4.37	16.23	44.12
Wood + Gypsum + Blue paint layer	S*	34.37	− 8.97	15.46	–	34.37	− 8.97	15.46	–	34.37	− 8.97	15.46	–
	E	32.35	− 11.88	− 10.91	26.60	17.37	− 5.13	− 8.32	24.41	36.86	− 7.77	− 6.70	22.33
Wood + Gypsum + Gilded layer	S*	73.09	6.29	34.70	–	73.09	6.29	34.70	–	73.09	6.29	34.70	–
	E	44.96	− 1.72	17.33	33.37	25.15	2.17	14.23	52.29	34.28	5.40	18.53	42.05

The typical stucco construction was built of pinewood, with a blue paint layer and gild coat covering the gypsum preparation layer. Comparing the discoloration to standard samples, a high degree was discovered. Fungal pigments may be responsible for these outcomes^83,84^. SEM and chromatic alterations were used to evaluate the biodeterioration symptoms of a wooden panel with a blue covering and a gild layer caused by *A. niger*, *A. flavus* and *A. terreus*.

#### Scanning Electron Microscope (SEM)

**Figure 7:**
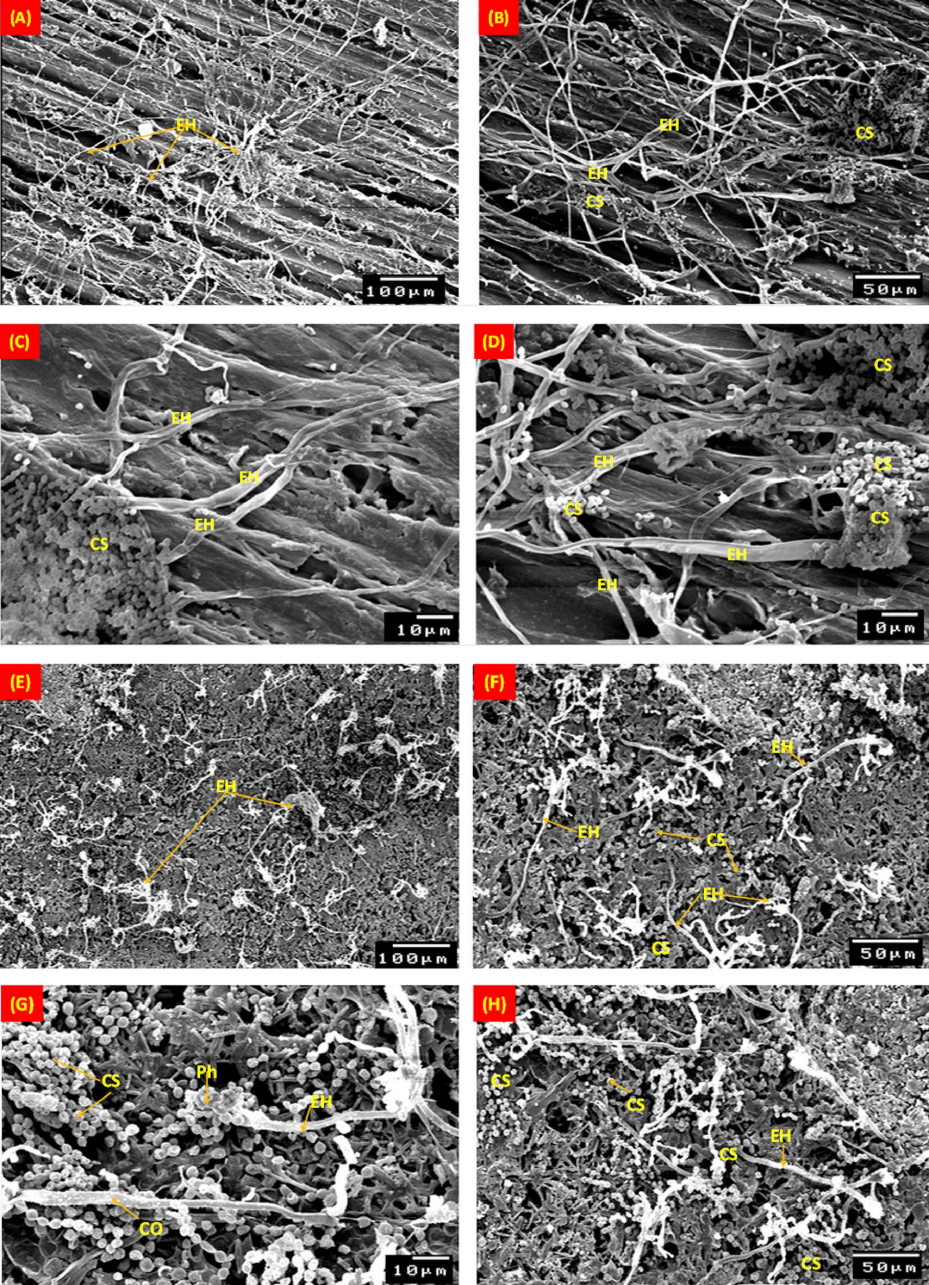

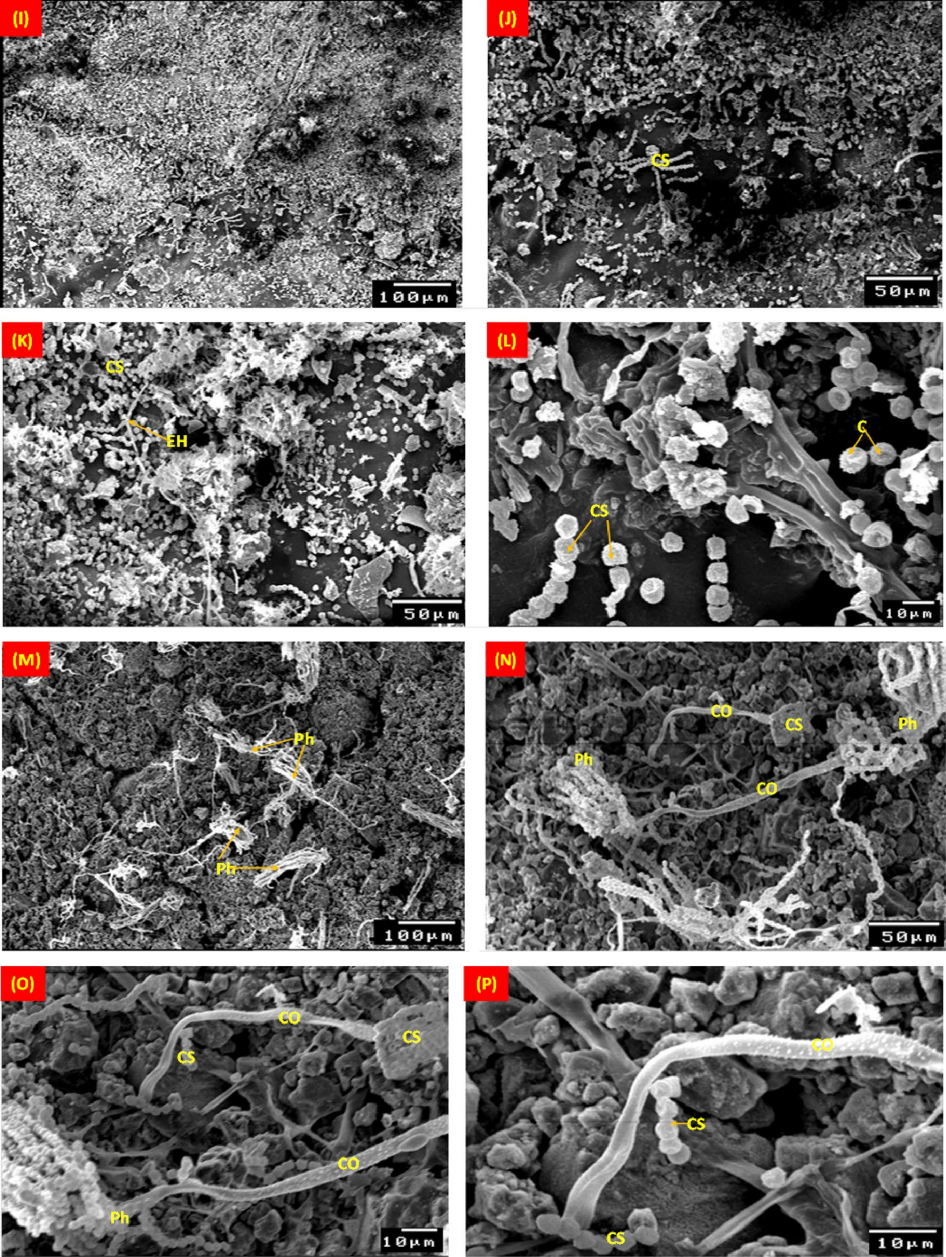
SEM micrographs of the mycelial morphology of *Aspergillus flavus* on pinewood (**A**–**D**), gypsum (**E**–**H**), gilded layer (**I**–**L**), and blue paint layer (**M**–**P**). EH: External hyphae; CS: Conidiospores; C: Conidia; CO: Conidiophores; Ph: Phialide.

Figure 7 displays SEM images of the *A. flavus* mycelial morphology colonized the structural layers of the typical stucco. External hyphae and the conidiospores were used to illustrate the growth spread of *A. flavus* on pinewood (Fig. 7A–D). Conidiophores also develop on the gypsum, and after six months of incubation, spores were visible on the surface (Fig. 7E–H). According to SEM images of *A. flavus* growth, spores instead of conidiophores separated from them on the gilded layer, and as a result, growth was reduced with the lack of nutrients (Fig. 7I–L). Due to copper's toxicity as the primary component of azurite, the blue coating layer (Azurite Cu_3_(CO_3_)_2_(OH)_2_), had less fungal colonization on the mycelial morphology of *A. flavus* (Fig. 7M–P).

**Figure 8:**
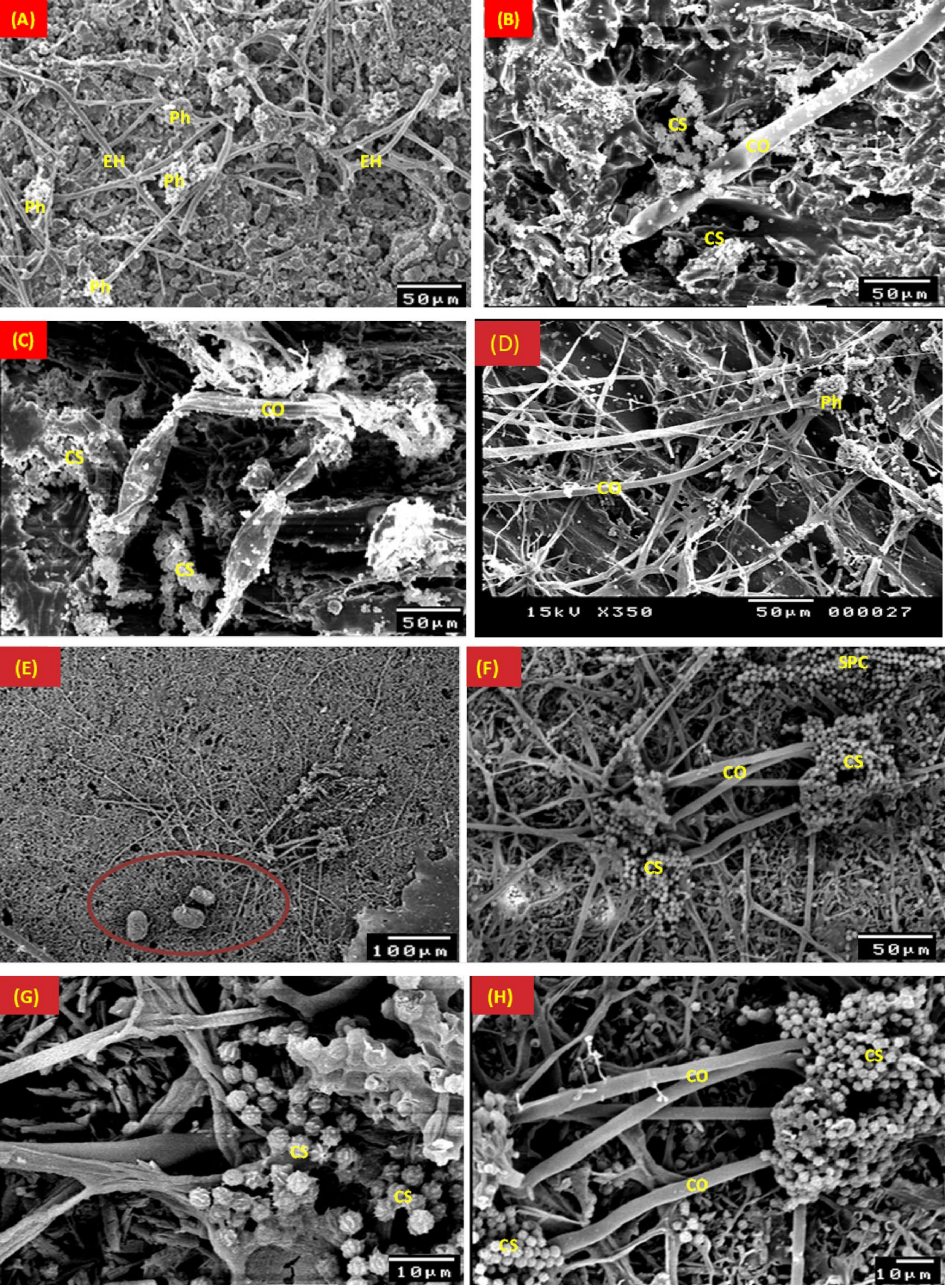

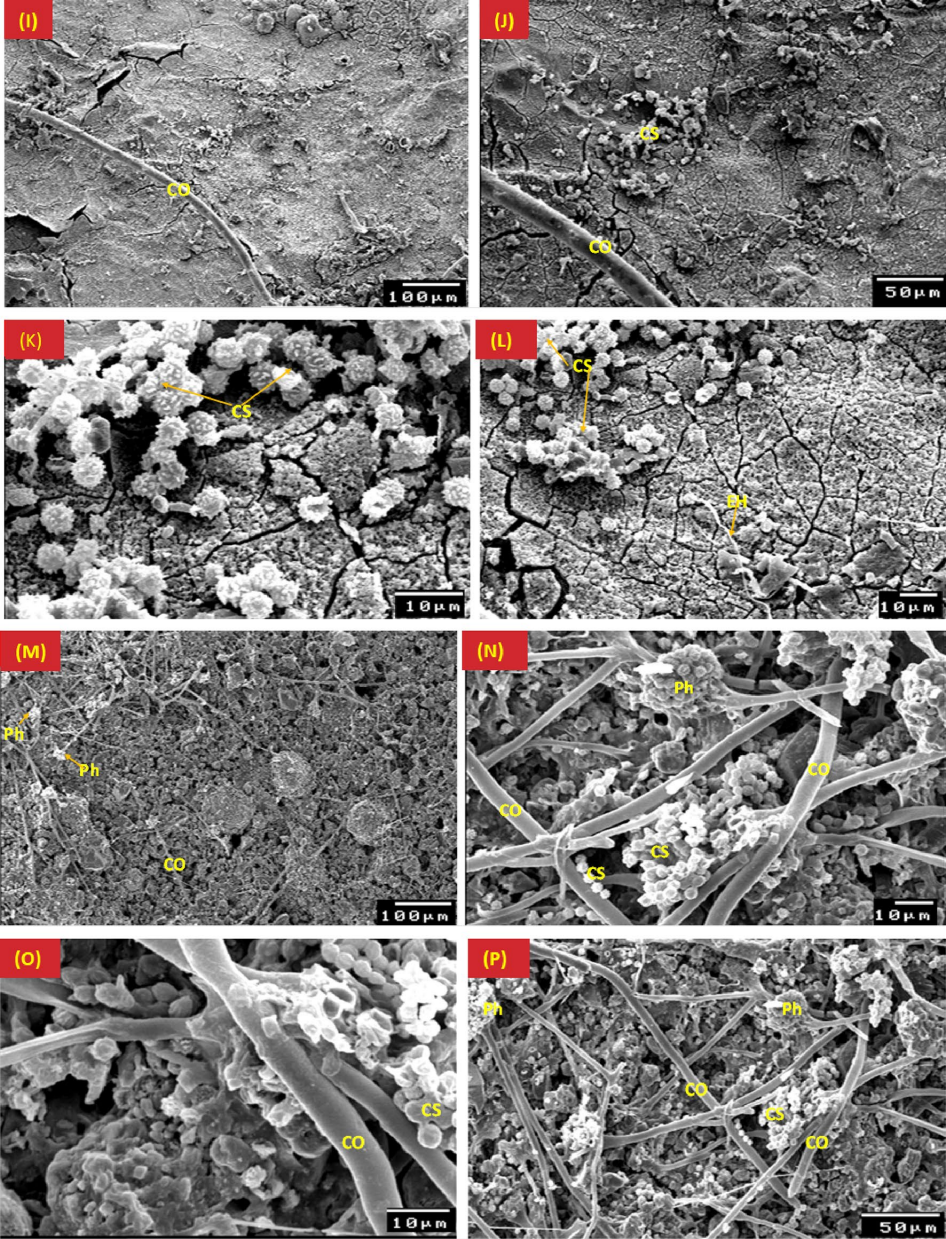
SEM micrographs of the *Aspergillus niger* mycelial growth on pinewood (**A**–**D**), gypsum (**E**–**H**), gilded layer (**I**–**L**) and blue paint layer (**M**–**P**). EH: External hyphae; CS: Conidiospores; C: Conidia; CO: Conidiophores; Ph: Phialide; circule refere to the fruiting growth of the fungus.

*A. niger* mycelial growth on the stucco model is shown in Fig. 8. Conidiospores were prominently displayed on pinewood and direct hyphal penetration into the wood was seen (Fig. 8A–D). After six months of incubation, the mycelial morphology of *A. niger* revealed the existence of conidia and conidiophores on the surface of gypsum, as well as the sexual reproduction, cleistothecia, through copulations that contained numerous ascospores (Fig. 8E–H). On gypsum's surface, cleistothecium production can be visible, while wood and gypsum both exhibit *A. niger* spores formation.

*A. niger* growth on the gilded layer revealed developed spores between the cracks of the layer, but after six months of incubation, these spores were spotted separately over the surface and covered the gilded layer (Fig. 8I–L). The *A. niger* mycelial morphology and growth density on the blue (Azurite) coating layer were depicted in the SEM micrographs (Fig. 8M–P).

**Figure 9:**
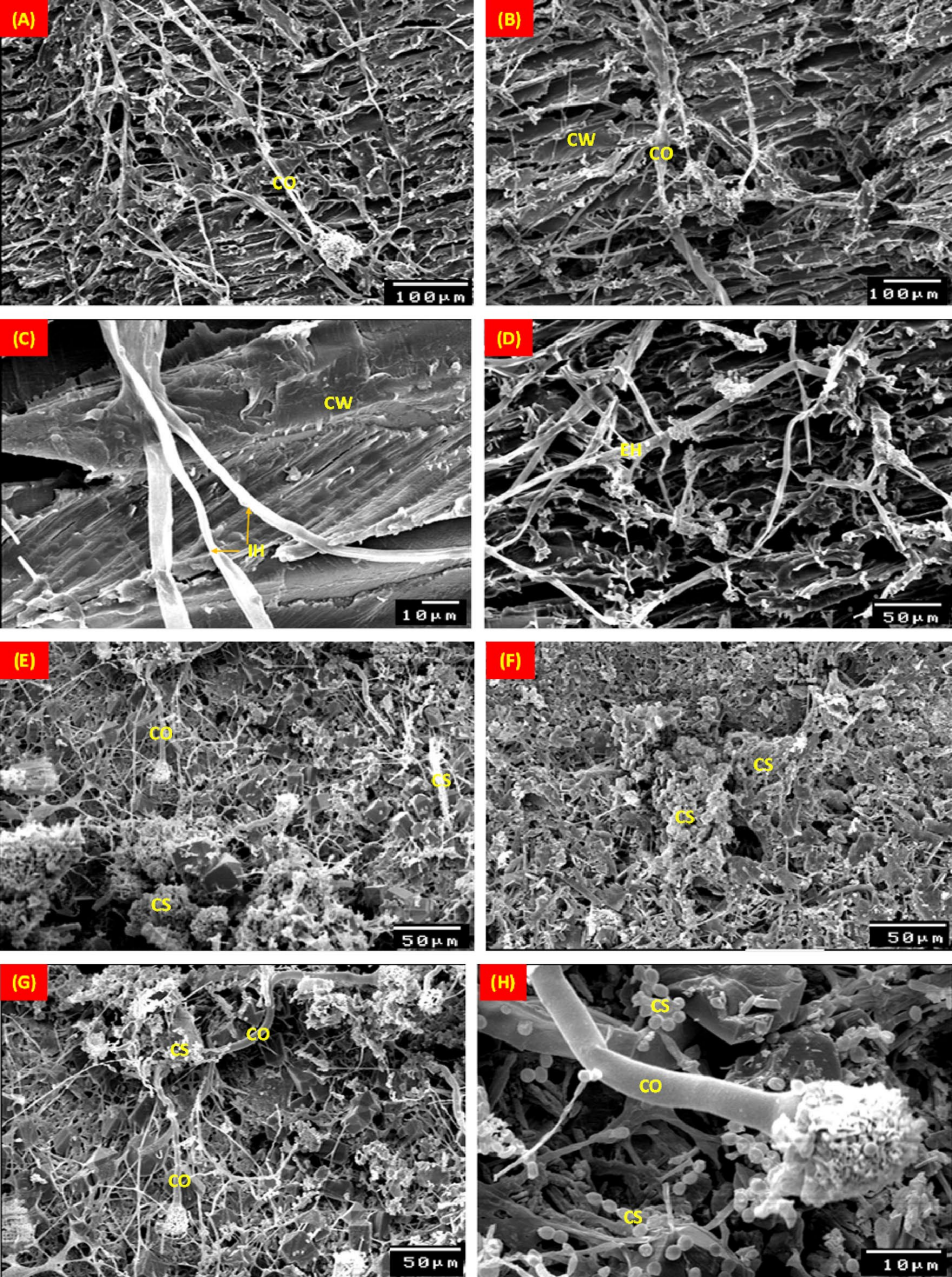

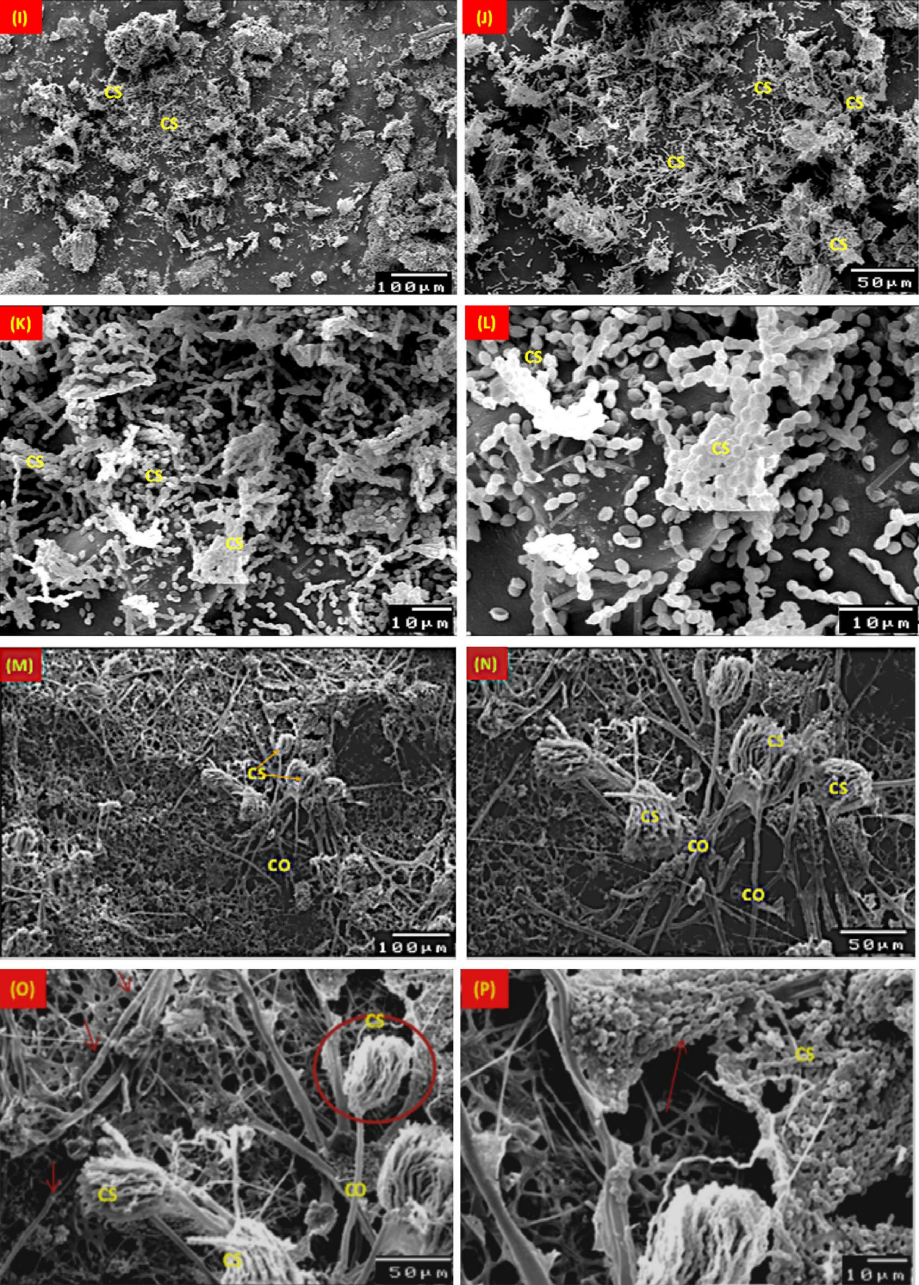
SEM micrographs of the mycelial morphology growth of *Aspergillus terreus* on pinewood (**A**–**D**), gypsum (**E**–**H**), blue gilded layer (**I**–**L**) and paint layer (**M**–**P**). EH: External hyphae; IH: Internal hyphae; CS: Conidiospores; C: Conidia; CO: Conidiophores; Ph: Phialide; CW: Cell wall.

Figure 9 displays SEM images of typical stucco layers colonized by *A. terreus*. *A. terreus* hyphae and spores proliferated quickly on the pinewood, where cavities seen in the primary and secondary layers of the pinewood (Fig. 9A–D). On gypsum, an *A. terreus* colony can be seen to be dense (Fig. 9E–H). _ENREF_65 *A. terreus* quickly spread its conidiophore and spores on the ground layer, where it feeds on gypsum as nutrition.

On the blue coating, the segmented filaments conidiophores of *A. terreus* are visible with large growths (Fig. 9I–L). The conidiospore-topped, branching hyphae were covered in fungal fruit bodies and produced spores that disseminated efficiently on wood and Azurite coating. In Fig.  9M–P, the growth of *A. terreus* on the gilded layer is exhibited. After 6-months of incubation, spores were visible on the surface and had completely covered the gilded layer.

It has been found that wood, wallpaper, and gypsum wall panels with cardboard face, as well as other textiles and painted surfaces, are suitable for fungi to colonize^85^. The relative humidity of building materials, such as gypsum, wood and concrete, affects the growth of fungi like *Cladosporium sphaerospermum*, *P. chrysogenum*, and *A. versicolor*^86–90^. Gypsum surfaces can support the growth of molds like *Chaetomium globosum* and *Stachybotrys chartarum*^91^.

*A. niger*, *Cladosporium halotolerans* and *Penicillium rubens* did not germinate on natural gypsum and flue-gas gypsum. In addition, *C. halotolerans* and *P. rubens* demonstrated growth and germination on phosphogypsum, although *A. niger* hardly germinated on this substrate^92^. On a gypsum substrate, *A. niger* spore production was found, on gypsum substrate, and obvious cleistothecium formation growth was demonstrated^93^. *A. niger* was raised on fiberglass, flower bed mulch, paint chips and wallboard^85^. *A. terreus* was able to penetrate the wood fiber surface and break the bundle, thus degrading the wood^94,95^.

We can see a thick, widespread fungal growth that has colonized over the wood panel support's gilded stucco framework. Previously, It has been previously noted that *A. niger*, *A. terreus*, *A. flavus*, and *A. humicola* have all been reported to have been found on degraded organic and inorganic antiquities traces in the excavations of Tuna el- Gabel's, Egypt^96^. *Aspergillus* spp. was found to be the most prevalent on all smears of damaged marble in three locations in Cairo, Egypt; Mohamed Ali palace, El-Ghory Mosque and Mosque of El-Kady Abdel-Baset in Cairo, Egypt^97^.

On the stone monuments of Dharmarajika, Taxila, Pakistan, Several fungi including *A. fumigatus*, *A. flavus*, and *A. niger*, were common and isolated from microbial decomposition, and their metabolic secretions (organic acids) were found on stone materials^98^. *A, niger*, *A. flavus*, *Acremonium cerealis*, and *Morterilla subtilssina* were found in the wall painting from the palace Mohamed Ali (1812) in Suez, Egypt, which had undergone biodeterioration^99^.

## Conclusion

The Sultan al-Ashraf Qaytbay Mausoleum in Egypt's Mamluk dynasty, with its gold-painted stucco decorations on a wooden panel, is the subject of the current work. Visual inspection revealed extensive biological deterioration. *Aspergillus* may grow on a wide variety of organic and inorganic materials, as is widely known. It may also grow in a variety of weather conditions. Factors related to the mosque's conditions contribute to the growth of fungi, which destroys the gilding layer and dissolves the paint and white ground layer. SEM–EDX, FTIR, and XRD spectroscopic investigations were also used to evaluate the painted and painted stucco decorations on the wood panel. The measurements revealed that the wooden panel is made of pinewood (*Pinus halepensis*), and the white ground layer of stucco was made of calcite as the main component with of traces of gypsum, coated with Azurite as a blue paint layer with a gilded coating layer as confirmed by EDX and XRD analyses. According to FTIR, animal glue served as the organic binding medium. To accurately assess the impact of the fungus infection by SEM analysis and colorimetric measurements, the mimic samples were designed using the exact same structures and materials as the actual object. Several symptoms are observed with widespread growth of fungi. After fungi invaded the stucco materials, a significant ΔE was observed. ΔE was found to be considerably high when compared to the reference samples. SEM and chromatic changes were used to evaluate the biodeterioration symptoms of the wooden panel with the blue paint layer and the gilded layer caused by *A. niger*, *A. flavus* and *A. terreus*. The prefabricated stucco structure, which was fully occupied, had a dense and broad growth of *A. terreus*. The pinewood and gypsum were colonized by *A. niger* and *A. flavus*, while the growth on the gilt layer is less noticeable. The results can be drawn to understand and the nature of the wooden panel and the impact of Aspergillosis on it, in order to design the most appropriate treatment plan for the chosen wooden panel to keep it from further damage in the future.

### Supplementary Information

Supplementary Figures.

## Data Availability

All data generated or analyzed during this study are included in this published article.
