# Supplementary material for: Biodeterioration effects of three Aspergillus species on stucco supported on a wooden panel modeled from Sultan al-Ashraf Qaytbay Mausoleum, Egypt
Source: Sci Rep. 2023 Sep 14;13:15241. doi: 10.1038/s41598-023-42028-x (PMC10502143; doi:10.1038/s41598-023-42028-x)
Supplement: Supplementary file 1 — Supplementary Figures. [file 41598_2023_42028_MOESM1_ESM.docx]

Biodeterioration effects of three *Aspergillus* species on stucco supported on a wooden panel modeled from Sultan al-Ashraf Qaytbay Mausoleum, Egypt

Hala A. M. Afifi ^1,*^, Maisa M. A. Mansour ^1,*^, Alyaa G. A. I. Hassan ^1^, Mohamed Z. M. Salem ^2,^*

*1 Conservation Department, Faculty of Archaeology, Cairo University, Giza 12613, Egypt;* [Halaafifi11@cu.edu.eg](mailto:Halaafifi11@cu.edu.eg); [maisamansour@cu.edu.eg](mailto:maisamansour@cu.edu.eg); catmashmash@yahoo.com

*2 Forestry and Wood Technology Department, Faculty of Agriculture (El-Shatby), Alexandria University, Alexandria 21545, Egypt; mohamed-salem@alexu.edu.eg*

Correspondence should be addressed to

Hala A. M. Afifi; [Halaafifi11@cu.edu.eg](mailto:Halaafifi11@cu.edu.eg)

Maisa M. A. Mansour; [maisamansour@cu.edu.eg](mailto:maisamansour@cu.edu.eg)

Mohamed Z. M. Salem; [mohamed-salem@alexu.edu.eg](mailto:mohamed-salem@alexu.edu.eg)

Fig. S1. EDX analysis for elemental composition of ground layer from the first point (a), second point (b), third point (c), fourth point (d), fifth point (e) and sixth point (f).

Fig. S2. EDX elemental analysis of blue pigment.

Fig. S3. EDX elemental analysis of gilded layer. (a) point 1 and (b) point 2.


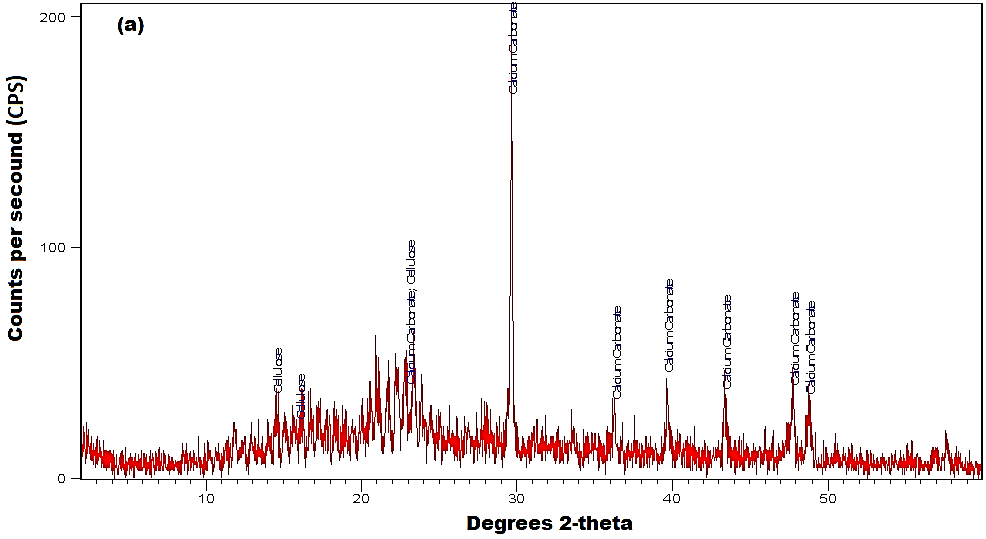


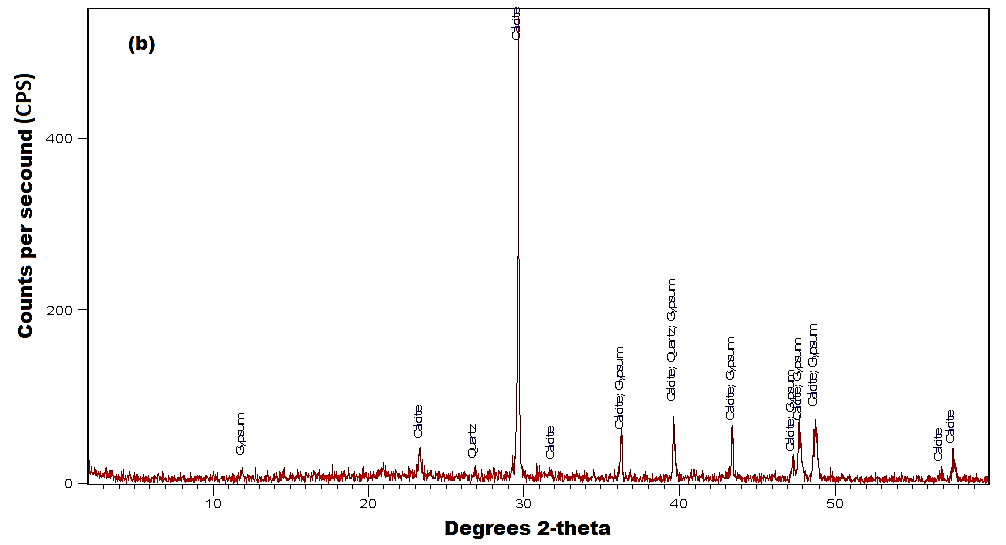


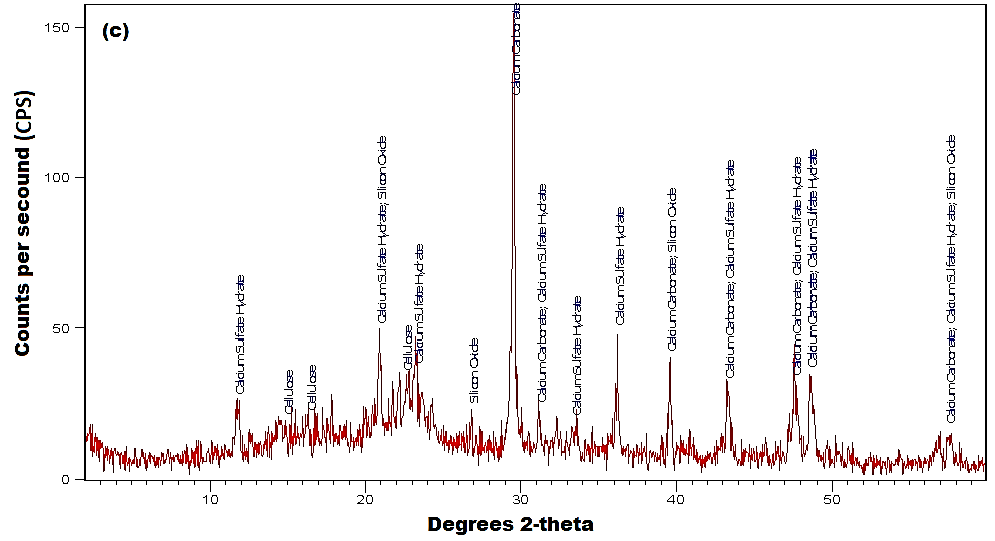


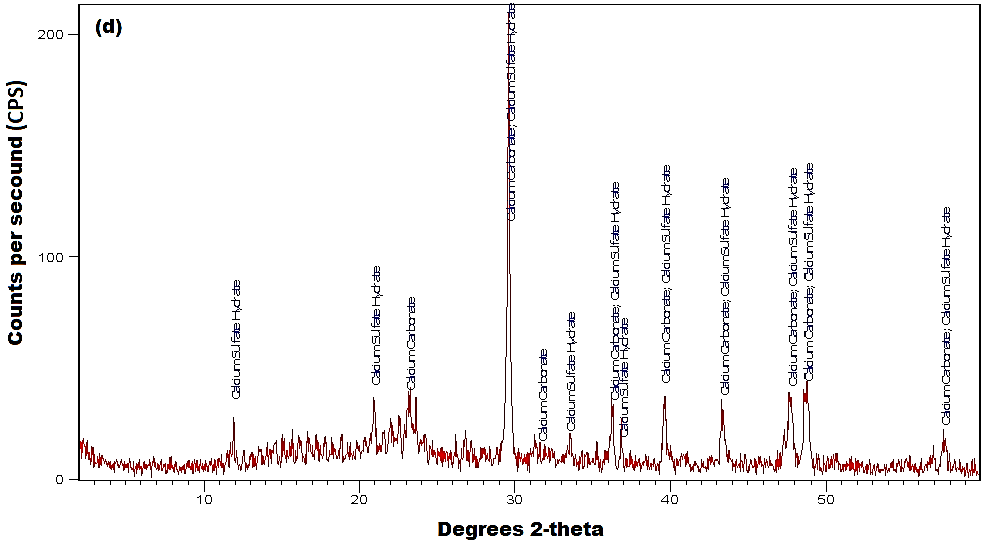


Fig. S4. XRD pattern of the analysis of the ground layer and wooden support, a-b: the XRD pattern of the ground preparation layer, c-d : the XRD pattern of the wooden support.
